# The Impact of Symptoms of Benign Anorectal Disorders on Self-Esteem and the Severity of Depression in Postpartum Women

**DOI:** 10.3390/jcm15135018

**Published:** 2026-06-27

**Authors:** Natalia Kuciel, Dominik Marciniak, Roma Roemer-Ślimak, Karolina Biernat, Justyna Mazurek, Edyta Sutkowska

**Affiliations:** 1 Department of Clinical Physiotherapy and Rehabilitation, University Center of Physiotherapy and Rehabilitation, Faculty of Physiotherapy, Wrocław Medical University, 50-368 Wrocław, Poland; natalia.kuciel@umw.edu.pl (N.K.); karolina.biernat@umw.edu.pl (K.B.); edyta.sutkowska@umw.edu.pl (E.S.); 2 Department and Division of Pharmaceutical Technology, Faculty of Pharmacy, Wrocław Medical University, 50-556 Wrocław, Poland; dominik.marciniak@umw.edu.pl; 3 Department and Division of Family Medicine, Faculty of Medicine, Wrocław Medical University, 51-141 Wrocław, Poland

**Keywords:** anorectal disorders, self-esteem, depression, postpartum women

## Abstract

**Background/Objective:** Anorectal disorders are a common yet underdiagnosed component of pelvic floor dysfunction, particularly among postpartum women. These conditions, including hemorrhoids, anal fissures, and fecal incontinence, are associated with physical discomfort and may negatively affect psychological well-being. Self-esteem has been identified as a potential mediator linking somatic symptoms to mental health outcomes, including depression. We aimed to assess the relationship between anorectal symptoms, self-esteem and the severity of depressive symptoms in a Polish population of postpartum women. **Methods:** A cross-sectional questionnaire-based study was conducted between June and December 2025, including 120 women aged >18 years, at least 3 months postpartum. Data were collected using online and paper questionnaires. The assessment tools included the Pelvic Floor Distress Inventory—Short Form 20 (PFDI-20), Rosenberg Self-Esteem Scale (RSES), and Beck Depression Inventory (BDI). Statistical analyses included the multiple regression model, logistic regression and Spearman’s rank correlation. **Results:** The most frequently reported anorectal symptoms included pain (45.83%), bleeding (42.5%), and pruritus (41.7%). The mean PFDI-20 score was 41.15 (SD 31.13). High self-esteem was observed in 55% of participants, whereas depressive symptoms were present in varying degrees of severity, ranging from no depressive symptoms (55.83%), mild (30%) to severe (0.83%). Significant positive correlations were found between pelvic floor dysfunction severity (particularly CRADI-8 and UDI-6 subscales) and depressive symptoms (BDI), while their correlation with self-esteem (RSES) was negative. The strongest positive correlation was observed between the overall PFDI-20 score and depression (r = 0.389). Multiple regression analyses identified anorectal symptoms, parity, hypothyroidism, and pre-pregnancy depression or anxiety as significant predictors of self-esteem. Anorectal symptoms and hormonal contraceptive use were significant predictors of depressive symptom severity. Among women who delivered vaginally, perineal tear was independently associated with greater depressive symptom severity (OR = 1.07, 95% CI: 1.01–1.14, *p* = 0.019). **Conclusions:** Postpartum anorectal and pelvic floor dysfunction symptoms are significantly associated with poorer psychological outcomes, including higher depressive symptoms and lower self-esteem. These findings highlight the importance of a multidisciplinary approach with routine psychological screening in postpartum care.

## 1. Introduction

Anorectal disorders constitute a significant, yet often underdiagnosed, component of pelvic floor dysfunction. Similar to urinary incontinence, the prevalence of anorectal conditions increases with age, and they share common risk factors such as pregnancy, obstetric trauma, and weakening of the pelvic floor muscles. Hemorrhoids are by far the most common proctologic condition, with a prevalence estimated at approximately 25–40% among pregnant and postpartum women, particularly in the third trimester of pregnancy and during the first weeks of the puerperium [1]. Anal fissure occurs significantly less frequently, with a prevalence of approximately 1–5%; however, in clinical studies involving proctologic examination, its detection rate may reach several to over a dozen percent [2,3]. The prevalence of fecal incontinence ranges from 6.8% to 9.2%, with higher rates observed in women and older individuals [4].

Anorectal disorders during pregnancy and after childbirth may result from the interaction of several key mechanisms: (1) mechanical: increased intra-abdominal pressure and venous pressure leading to hemorrhoids, (2) hormonal: progesterone causes slowed peristalsis and constipation, (3) traumatic: stretching and damage to the pelvic floor muscles and anal sphincter (OASI), (4) neurological: possible pudendal nerve neuropathy during childbirth, and (5) functional: defecation disorders and weakened sphincter control [5,6].

Self-esteem is an important component of psychological well-being and reflects an individual’s overall sense of self-worth and value [7]. Lower self-esteem has consistently been associated with poorer mental health outcomes, including greater severity of depressive symptoms and increased vulnerability to postpartum depression [8]. Anorectal disorders are frequently accompanied by chronic pain, discomfort, bleeding, pruritus, and functional impairment, which may negatively affect daily activities, social functioning, and body perception. In postpartum women, symptoms such as fecal or flatal incontinence may additionally evoke feelings of embarrassment, loss of bodily control, and social withdrawal [9,10,11]. These psychosocial consequences may adversely affect self-esteem and contribute to emotional distress. Previous studies have shown that patients with hemorrhoidal disease and other anorectal disorders report reduced quality of life, impaired psychological well-being, and more frequent depressive symptoms [9,10,12]. However, the relationship between anorectal symptoms, self-esteem, and depression in postpartum women remains insufficiently explored. Given the established associations between physical symptoms, self-esteem, and mental health, the aim of this study was to assess the relationship between anorectal symptoms, self-esteem, and depressive symptom severity among Polish postpartum women.

## 2. Materials and Methods

This study based on a cross-sectional questionnaire was conducted between June and December 2025. The Bioethics Committee of Wroclaw Medical University decided that the project was not a research experiment and did not require the committee’s approval to conduct the study (decision no. 161/2025).

### 2.1. Inclusion and Exclusion Criteria

Inclusion criteria: vaginal delivery or caesarean section, >3 months after delivery (period of tissue healing after delivery), age > 18 years.

Exclusion criterion: no births, age < 18 years.

### 2.2. Questionnaires

The following questionnaires were used in the study: a self-developed sociodemographic, clinical, and obstetric questionnaire (details regarding the information collected using this questionnaire are presented in Table 1), the Pelvic Floor Distress Inventory–Short Form 20 (PFDI-20), the Rosenberg Self-Esteem Scale (RSES), and the Beck Depression Inventory (BDI).

Incomplete responses to any of the primary outcome questionnaires (PFDI-20, RSES, or BDI) constituted grounds for exclusion from the analysis. In contrast, occasional missing data in the self-developed sociodemographic, clinical, and obstetric questionnaire did not result in exclusion and were retained in the dataset. The frequency of such missing data is reported in Table 1.

The questionnaires were distributed in the following form:

1. Online: Social media on urogynecological and perinatal closed groups. After obtaining the administrator’s consent, an invitation to the survey was posted along with a link. Anonymous individuals filled out the questionnaire and sent their responses. For our purposes, the questionnaire was adapted to an online version using the Google module.
2. By inviting patients in 3 selected gynaecological and general medical clinics to participate in the study and fill out paper questionnaires.

In total, 180 questionnaires were distributed in the clinics. A total of 128 responses were obtained, including 75 online and 53 paper questionnaires.

Eight questionnaires were excluded from the statistical analysis due to incomplete responses in the PFDI-20, RSES, or BDI questionnaires. Missing data were limited to selected baseline characteristics and occurred infrequently.

Informed consent was obtained. For paper questionnaires, acceptance of the anonymous questionnaire was regarded as consent, whereas for online questionnaires, completion of the questionnaire was considered as consent to anonymous participation in the study.

The Pelvic Floor Distress Inventory—Short Form 20 (PFDI-20) [13] questionnaire was used to assess the symptoms of pelvic floor dysfunction. It is a standardized self-reporting tool consisting of 20 questions divided into three subscales: POPDI-6 (pelvic organ prolapse symptoms), CRADI-8 (gastrointestinal symptoms), and UDI-6 (urinary symptoms). Patients rate the presence and severity of symptoms on a scale of 0–3, and the results of the subscales are in the range of 0–100 points; the sum of the points of all subscales forms the total PFDI-20 score (0–300 points), where higher values indicate greater severity of symptoms. The PFDI-20 questionnaire is a widely used tool in research on pelvic floor dysfunction, and its psychometric properties have been confirmed in many female populations. The tool is characterized by good reliability and validity and allows for the assessment of both the frequency of symptoms and their impact on daily life.

The Rosenberg Self-Esteem Scale (RSES) [14] is a tool used to assess an individual’s overall self-esteem. It consists of 10 items referring to the general attitude towards each other, including both positive and negative statements. Responses are given on a four-point Likert scale, and the result obtained reflects the level of self-assessment, where higher values indicate a more positive assessment of one’s own person. The scale is characterized by high reliability and good validity and is widely used in research on mental health, including the analysis of the relationship between self-esteem and depression.

The Beck Depression Inventory (BDI) [15] is one of the most commonly used tools for assessing the severity of depressive symptoms. It consists of 21 items referring to various aspects of mental and somatic functioning, such as mood, guilt, energy level, or sleep disorders. Each item is rated on a four-point scale from 0 to 3 points, which allows for a total score of 0 to 63 points. Higher results indicate a greater severity of depression symptoms. The scale is characterized by high reliability and validity and is widely used in both scientific research and clinical practice, including the assessment of the mental health of women in the postpartum period.

### 2.3. Statistics

The variables included in the statistical analysis were both nominal—including dichotomous—and continuous. Nominal variables were characterized using frequency tables, which additionally presented the percentage contribution of each category within the analysed variable. For continuous variables measured on ratio scales, basic descriptive statistics were calculated, including the mean, standard deviation, median, and range. The normality of the distribution of continuous variables was assessed using the Shapiro–Wilk W test. If the variables were not normally distributed, non-parametric correlation analyses were used where appropriate.

The results of the psychological assessments were continuous variables. The degree of correlation between them was evaluated using Spearman’s rank-order correlation matrices, along with tests of statistical significance.

The dependent variables were dichotomous in nature; therefore, the primary statistical method used in this study was logistic regression. The results were presented as odds ratios (OR), along with their statistical significance and 95% confidence intervals (OR ± 95% CI).

To evaluate the associations between the scores obtained from the PFDI-20, RSES, and BDI, treated as continuous outcome variables, and the following explanatory variables: presence of anorectal symptoms, rectal bleeding, anal itching, anal pain, parity, mode of delivery, physical activity before and during pregnancy, comorbid conditions (including low back pain, headache and neck pain, inflammatory bowel disease, previous abdominal surgery, diagnosed depression or anxiety disorder, hypothyroidism), and hormonal contraceptive use, multiple linear regression analysis was performed using a model without an intercept term (β_0_ = 0). Residual analysis was performed for the multivariable regression models to assess model assumptions and the adequacy of fit. The normality of residuals was evaluated using the Shapiro–Wilk W test.

In all statistical analyses, a significance level of α = 0.05 was adopted. Analyses were performed using STATISTICA PL^®^ v. 13.

## 3. Results

A total of 128 questionnaires were received. Eight questionnaires were excluded due to incomplete responses in the PFDI-20, RSES, and/or BDI questionnaires, leaving 120 participants for the final analysis. Because complete data were available for all variables required to address the study objectives and test the research hypotheses, sporadic missing values did not compromise the validity of the analyses and were not considered to have a meaningful impact on the study outcomes.

The characteristics of the study group are presented in Table 1 and organized into thematic categories.

Among women who delivered vaginally (66.67%), 15.83% had a spontaneous delivery without medical intervention, 35% received oxytocin, and 19.17% underwent other interventions (Instrumental or Kristeller maneuver). Cesarean section was performed in 31.67% of the study population.

The most commonly reported anorectal symptoms were pain (45.83%), bleeding (42.5%), and pruritus (41.7%).

The mean score of the PFDI-20 questionnaire for the entire study group was 41.15 (SD 31.13) points. The mean scores for the subscales were as follows: (1) POPDI-6—9.7 (SD 12.51); (2) CRADI-8—17.75 (SD 14.98); (3) UDI-6—13.69 (SD 15.95).

According to the RSES, 66 women (55%) had high self-esteem, 26 (21.67%) had moderate self-esteem, and 28 (23.33%) had low self-esteem.

Based on the BDI, no depressive symptoms were observed in 67 women (55.83%), mild symptoms in 36 (30%), moderate symptoms in 16 (13.33%), and severe depression in 1 participant (0.83%).

Statistically significant correlations were found between all subscales of the PFDI-20, indicating co-occurrence of pelvic floor dysfunction symptoms. Greater severity of anorectal symptoms (CRADI-8) and urinary symptoms (UDI-6) was associated with higher levels of depression and lower self-esteem among postpartum women (Table 2).

The correlation between RSES and BDI was −0.6113 and was statistically significant.

Multiple linear regression analysis for RSES demonstrated that presence of anorectal symptoms (ꞵ = −0.4315, 95% CI: −0.5925–−0.2705, *p* = 0.000002), parity (ꞵ = 0.1916, 95% CI: 0.0552–0.3281, *p* = 0.0068), hypothyroidism (ꞵ = 0.2296, 95% CI: 0.0916–0.3676, *p* = 0.0015), a pre-pregnancy diagnosis of depression or anxiety disorder (ꞵ = 0.1860, 95% CI: 0.0297–0.3423, *p* = 0.0206) were significant predictors of self-esteem scores. The overall regression model was statistically significant (Multiple R = 0.913, *p* = 3.77 × 10^−14^) (Table 3).

Multiple linear regression analysis for BDI revealed that the presence of anorectal symptoms (ꞵ = −0.3077, 95% CI: −0.5767–0.0386, *p* = 0.0258) and the use of hormonal contraception (ꞵ = 0.2691, 95% CI:0.0198–0.5183, *p* = 0.0348) were significant predictors of depressive symptom severity. The overall model was statistically significant (Multiple R = 0.730, *p* = 0.00028) (Table 4).

Logistic regression analysis was performed to evaluate the effects of episiotomy and perineal tear on BDI and RSES outcomes. Among women who experienced vaginal delivery, perineal tear was significantly associated with greater severity of depressive symptoms (OR = 1.07, 95% CI: 1.011–1.135, *p* = 0.019).

## 4. Discussion

The postpartum period is associated with substantial physical and psychological changes that may increase vulnerability to mental health problems. Anorectal disorders, including fecal incontinence and hemorrhoidal disease, may further be associated with psychological distress through embarrassment, impaired bodily control, and limitations in daily functioning. Therefore, the consequences of these conditions should be considered not only from a somatic perspective but also within a broader biopsychosocial framework [16].

Previous studies have demonstrated that obstetric perineal trauma and obstetric anal sphincter injuries (OASI) are associated with poorer psychological outcomes after childbirth, including increased depressive symptoms and reduced quality of life [17,18]. Persistent anorectal symptoms, chronic perineal pain, and functional impairment are also associated with reduced maternal well-being and psychosocial functioning. These findings support the concept that pelvic floor and anorectal disorders are associated with adverse mental health outcomes during the postpartum period [18].

Similar relationships between pelvic floor dysfunction and psychological outcomes have also been observed in European populations. A prospective cohort study conducted in France showed that postpartum urinary incontinence was associated with higher depressive symptom severity measured by the Edinburgh Postnatal Depression Scale (EPDS) at both 4 and 12 months after delivery, as well as increased use of antidepressant medication, suggesting a persistent impact on maternal mental health over time [19]. In addition, a meta-analysis including European and international studies reported a significant association between urinary incontinence and postpartum depression (pooled OR ≈ 1.45), indicating a consistent but moderate increase in the risk of depressive symptoms in affected women [20]. Population-based studies from Western Europe have further demonstrated that both urinary and anal incontinence in the postpartum period are associated with reduced health-related quality of life and higher levels of depressive symptoms [21]. These findings are consistent with evidence from broader pelvic floor dysfunction cohorts, which also highlight impaired psychosocial functioning and reduced emotional well-being in affected women [22].

In this context, evidence from different studies suggests that hypothyroidism is associated with increased risk of depressive and anxiety symptoms and reduced quality of life; however, its relationship with self-esteem remains poorly investigated and is likely indirect, mediated by affective symptoms and overall psychological burden [23,24]. Similarly, pre-pregnancy depression and anxiety have been consistently associated with poorer postpartum mental health outcomes and lower self-worth, although the strength of this relationship may vary depending on clinical and psychosocial factors [25]. Thus, the positive associations observed in the present study may reflect residual confounding, health-care engagement, or sample-specific characteristics rather than a direct causal effect.

In addition, residual confounding cannot be excluded, as several potentially important postpartum factors were not assessed. Variables such as breastfeeding status, marital status, sleep disturbances, psychiatric treatment, and psychosocial support are known to be associated with postpartum mental health and may influence maternal self-esteem; therefore, their absence from the analyses may have affected the observed associations [26].

In the present study, we analyzed associations between pelvic floor dysfunction symptoms assessed using the PFDI-20 questionnaire and psychological outcomes measured with BDI and RSES. The highest correlations were observed for the CRADI-8 subscale, which evaluates colorectal and anal symptoms.

We found that greater severity of anorectal symptoms was significantly associated with both higher levels of depressive symptoms and lower self-esteem. These findings are consistent with previous reports indicating that bowel dysfunction and fecal incontinence are associated with poorer psychological well-being, reduced quality of life, and increased risk of depression [27,28]. Women experiencing anorectal symptoms frequently report social embarrassment, avoidance behaviors, and diminished confidence in daily activities, which are associated with their psychological functioning.

Importantly, our findings extend the existing literature by demonstrating a relationship not only with depressive symptoms but also with self-esteem. While depression reflects current emotional distress, self-esteem represents an individual’s overall evaluation of self-worth. The observed association between CRADI-8 scores and lower self-esteem suggests that anorectal symptoms are associated with psychological functioning through mechanisms related to shame, loss of bodily control, and negative self-perception. Given the stigmatizing nature of anorectal disorders, these symptoms may be associated with lower self-esteem even beyond their immediate emotional state [29].

These observations are consistent with recent evidence indicating that self-esteem is a key psychological construct associated with postpartum mental health. A cross-sectional study conducted in Romanian postpartum women using the RSES demonstrated that lower self-esteem is significantly associated with higher levels of both depressive and anxiety symptoms, suggesting that global self-worth is closely linked to psychological well-being during the postpartum period. These findings support the role of self-esteem as an important intermediary factor in postpartum mental health and further reinforce the relevance of including self-perception measures when assessing women with pelvic floor dysfunction [12].

As the study was conducted using a self-administered questionnaire, all data were self-reported and were not verified through physical examination or objective clinical assessment. Consequently, the findings may be influenced by reporting bias, recall bias, and potential misclassification of anorectal symptoms. Given the sensitive nature of anorectal and pelvic floor dysfunction symptoms, which may be associated with embarrassment and limited awareness of their clinical significance, the use of an anonymous online questionnaire may have reduced reporting barriers and encouraged more candid responses. This aspect was highlighted in the study invitation and participant information sheet.

Overall, our results indicate that postpartum anorectal symptoms are associated with both increased depressive symptom severity and reduced self-esteem. These findings highlight the importance of routine assessment of anorectal complaints after childbirth and support the integration of psychological screening into postpartum care for women experiencing pelvic floor dysfunction.

## 5. Conclusions

Postpartum anorectal and pelvic floor dysfunction symptoms are associated with worse psychological outcomes, including higher depressive symptom severity and lower self-esteem.

Anorectal symptoms (CRADI-8) show significant associations with both depressive symptom severity and self-esteem, suggesting a relationship between colorectal/anal symptom burden and psychological functioning.

Pelvic floor dysfunction is associated with both somatic symptoms and psychosocial outcomes, including measures of emotional well-being and self-perception in postpartum women.

Depressive symptom severity is associated with the current psychological burden reported in women with pelvic floor dysfunction, whereas lower self-esteem is associated with more general evaluations of self-worth in this population.

The findings support the relevance of a biopsychosocial approach in the assessment of postpartum women with pelvic floor disorders, considering both physical symptoms and psychological measures.

Psychological screening may be particularly relevant in women reporting anorectal symptoms, as these symptoms are associated with differences in depressive symptom levels and self-esteem scores.

A multidisciplinary approach integrating urogynecological and psychological care may be considered in the management of women with pelvic floor dysfunction to address both physical and psychological aspects of health-related quality of life.

### 5.1. Limitations

The study has several limitations that should be considered when interpreting the results. First, the relatively small sample size, resulting from the short recruitment period as well as participant refusal and non-return of paper questionnaires, reduces statistical power and limits the generalizability of the findings. In addition, the exploratory analyses included a relatively large number of predictors in relation to the available sample size, which may have increased the risk of model overfitting and affected the stability of some regression estimates. Consequently, the observed associations require confirmation in larger studies.

Another limitation is the reliance on self-reported data without objective clinical verification. No confirmatory diagnostic methods, such as ultrasound examination, anorectal manometry, or clinical assessment scales, were systematically applied. This may have introduced reporting bias and may have affected the accuracy of symptom severity assessment.

A further limitation is the lack of confirmed medical diagnoses in a proportion of participants, as analyses were largely based on self-reported anorectal symptoms. It should also be emphasized that anorectal and pelvic floor disorders in the postpartum period are frequently underdiagnosed, partly due to underreporting related to embarrassment and partly due to limited clinical attention to mild symptoms.

Importantly, the study did not control for all potential conditions that may influence symptom reporting. In particular, dermatological and allergic conditions that may cause perianal itching (pruritus ani), such as skin diseases or allergic reactions, were not systematically excluded. This may have introduced additional confounding in the assessment of anorectal symptom burden, especially in relation to CRADI-8 scores.

In addition, residual confounding cannot be excluded, as several potentially important postpartum factors were not assessed. Variables such as breastfeeding status, marital status, sleep disturbances, psychiatric treatment, social support, and other psychosocial factors may influence maternal self-esteem and psychological well-being. Because these variables were not collected and therefore could not be included in the analyses, they may have affected the observed associations.

Finally, the cross-sectional design of the study precludes any inference regarding temporal or causal relationships between pelvic floor dysfunction symptoms and psychological outcomes.

### 5.2. Clinical Implications

The observed associations between CRADI-8 scores, overall pelvic floor dysfunction symptom severity, depressive symptoms, and self-esteem highlight the importance of a multidisciplinary approach in the management of women with pelvic floor disorders.

In clinical practice, routine screening for psychological symptoms may be particularly relevant in patients reporting bowel and proctological complaints, given their association with emotional well-being and self-perception measures.

Integration of urogynecological and psychological care may support a more comprehensive assessment of patient needs. Such an approach may facilitate earlier identification of women experiencing psychological distress and support appropriate referral for further evaluation.

Additionally, greater attention to differential diagnosis of anorectal symptoms, including potential dermatological or allergic causes of perianal itching, may improve clinical accuracy in symptom interpretation.

## Figures and Tables

**Table 1:** Baseline characteristics of the study population.

Demographic Characteristics		Mean (SD)
Age [years]		37.11 (8.75)
Height [cm]		166 (6.17)
**Socioeconomic characteristics**		**N (%)**
Place of residence	Rural	30 (25)
	Urban	28 (23.33)
	Metropolitan	61 (5.83)
	Missing data *	1 (0.83)
Type of occupation	Sedentary	66 (55)
	Physical active	53 (44.17)
	Missing data *	1 (0.83)
Education	Higher education	101 (84.17)
	Secondary and primary education	18 (15)
	Missing data *	1 (0.83)
**Clinical/obstetric characteristics**		**Mean (SD) or N (%)**
Pre-pregnancy body weight [kg]		61.14 (9.38)
Pre-pregnancy BMI [kg/m^2^]		22.06 (2.89)
Body weight at the end of pregnancy [kg]		75.73 (11.05)
BMI at the end of pregnancy [kg/m^2^]		27.33 (3.28)
Current body weight [kg]		65.33 (9.55)
Current BMI [kg/m^2^]		23.61 (3.07)
Number of deliveries	One	62 (51.67)
	Two	44 (36.67)
	Three	10 (8.33)
	Missing data *	4 (3.33)
Mode of delivery	Cesarean section	38 (31.67)
	Vaginal delivery	80 (66.67)
	Missing data *	2 (1.67)
Time since delivery	<6 months	47 (39.17)
	>6 months	69 (57.50)
	Missing data *	4 (3.33)
Vaginal delivery without any intervention		19 (15.83)
Vaginal delivery with medical intervention (Instrumental or Kristeller maneuver)		23 (19.17)
Oxytocin		42 (35)
Episiotomy		40 (33.33)
Vaginal tear		58 (48.33)
**Comorbidities**		**N (%)**
Inflammatory bowel diseases and irritable bowel syndrome		26 (21.67)
Low back pain		42 (34.17)
Neck or back pain and headaches		33 (27.50)
History of abdominal and anorectal surgery		31 (25.83)
Depression and anxiety disorders (before delivery)		19 (15.83)
Hypothyroidism		28 (23.33)
Contraception		21 (17.50)
**Lifestyle factors**		**N (%)**
Physical activity before pregnancy	Yes	82 (68.33)
	No	34 (28.33)
	Missing data *	4 (3.33)
Physical activity during pregnancy	Yes	71 (59.17)
	No	45 (37.50)
	Missing data *	4 (3.33)
**Outcome variables**		**N (%)**
Presence of at least one anorectal symptom	Yes	74 (61.67)
	No	44 (36.67)
Presence of at least one anorectal symptom	Pain	55 (45.83)
	Bleeding	51 (42.5)
	Pruritus	50 (41.67)
	Fecal incontinence	14 (11.67)
	Swelling	13 (10.83)
	Rectal prolapse	9 (7.5)

Legend: SD—standard deviation, N—number of participants, BMI—body mass index, missing data *—missing data were limited to selected baseline characteristics.

**Table 2:** Correlations between Pelvic Floor Distress Inventory-20 and the Rosenberg Self-Esteem Scale and the Beck Depression Inventory.

Questionnaire	RSES	BDI
PFDI-20 (UDI-6)	−0.1210	0.3350
PFDI-20 (CRADI-8)	−0.2489	0.3126
PFDI-20 (POPDI-6)	−0.1274	0.1670
PFDI-20 (total score)	−0.2329	0.3890

Legend: PFDI—Pelvic Floor Distress Inventory, UDI—Urinary Distress Inventory, CRADI-8—Colorectal-Anal Distress Inventory, POPDI—Pelvic Organ Prolapse Distress Inventory, RSES—Rosenberg Self-Esteem Scale, BDI—Beck Distress Inventory.

**Table 3:** Summary of the multiple linear regression analysis predicting RSES scores.

Dependent Variable	Multiple R	Multiple R^2^	Adjusted R^2^	SS Model	df Model	MS Residual	SS Model	df Residual	MS Residual	F	*p*
RSES	0.912567	0.832778	0.779267	48,459.37	16	3028.711	9730.627	50	194.6125	15.5627	3.77 × 10^−14^

Legend: RSES—Rosenberg Self-Esteem Scale; R—multiple correlation coefficient; R^2^—coefficient of determination; Adjusted R^2^—adjusted coefficient of determination; SS—sum of squares; df—degrees of freedom; MS—mean square; F—Fisher’s F-statistic; *p*—significance level.

**Table 4:** Summary of the multiple linear regression analysis predicting BDI scores.

Dependent Variable	Multiple R	Multiple R^2^	Adjusted R^2^	SS Model	df Model	MS Residual	SS Model	df Residual	MS Residual	F	*p*
BDI	0.730084	0.533023	0.383590	8347.132	16	521.6958	7312.868	50	146.2574	3.56697	0.000287

Legend: BDI—Beck Depression Inventory; R—multiple correlation coefficient; R^2^—coefficient of determination; Adjusted R^2^—adjusted coefficient of determination; SS—sum of squares; df—degrees of freedom; MS—mean square; F—Fisher’s F-statistic; *p*—significance level.

## Data Availability

The original contributions presented in this study are included in the article. Further inquiries can be directed to the corresponding author.
